# Automated PGP9.5 immunofluorescence staining: a valuable tool in the assessment of small fiber neuropathy?

**DOI:** 10.1186/s13104-016-2085-4

**Published:** 2016-05-23

**Authors:** Nathalie Van Acker, Michael Ragé, Ellen Sluydts, Michiel W. M. Knaapen, Martine De Bie, Maarten Timmers, Erik Fransen, Carla Duymelinck, Stefanie De Schepper, Praveen Anand, Theo Meert, Léon Plaghki, Patrick Cras

**Affiliations:** Faculty of Medicine and Health Sciences, University of Antwerp, Antwerp, Belgium; HistoGeneX NV, Pr J Charlottelaan 10, Berchem, 2600 Antwerp, Belgium; Institute of Neuroscience, Université Catholique de Louvain, Avenue Mounier 53, B1.53.04, 1200 Brussels, Belgium; Janssen Research and Development, Janssen Pharmaceutica NV, Turnhoutseweg 30, 2340 Beerse, Belgium; StatUa Center for Statistics, University of Antwerp, Antwerp, Belgium; Peripheral Neuropathy Unit, Hammersmith Hospital, Du Cane Road, London, W12 0HS UK; Department of Neurology, Antwerp University Hospital, Born Bunge Institute, University of Antwerp, Wilrijkstraat 10, 2650 Edegem, Belgium; Reference Center for Biological Markers of Dementia (BIODEM), Institute Born-Bunge, University of Antwerp, Antwerp, Belgium

**Keywords:** Small fiber neuropathy, Skin biopsy, Diagnostic, Clinical, Immunofluorescence

## Abstract

**Background:**

In this study we explored the possibility of automating the PGP9.5 immunofluorescence staining assay for the diagnosis of small fiber neuropathy using skin punch biopsies. The laboratory developed test (LDT) was subjected to a validation strategy as required by good laboratory practice guidelines and compared to the well-established gold standard method approved by the European Federation of Neurological Societies (EFNS). To facilitate automation, the use of thinner sections. (16 µm) was evaluated. Biopsies from previously published studies were used. The aim was to evaluate the diagnostic performance of the LDT compared to the gold standard. We focused on technical aspects to reach high-quality standardization of the PGP9.5 assay and finally evaluate its potential for use in large scale batch testing.

**Results:**

We first studied linear nerve fiber densities in skin of healthy volunteers to establish reference ranges, and compared our LDT using the modifications to the EFNS counting rule to the gold standard in visualizing and quantifying the epidermal nerve fiber network. As the LDT requires the use of 16 µm tissue sections, a higher incidence of intra-epidermal nerve fiber fragments and a lower incidence of secondary branches were detected. Nevertheless, the LDT showed excellent concordance with the gold standard method. Next, the diagnostic performance and yield of the LDT were explored and challenged to the gold standard using skin punch biopsies of capsaicin treated subjects, and patients with diabetic polyneuropathy. The LDT reached good agreement with the gold standard in identifying small fiber neuropathy. The reduction of section thickness from 50 to 16 µm resulted in a significantly lower visualization of the three-dimensional epidermal nerve fiber network, as expected. However, the diagnostic performance of the LDT was adequate as characterized by a sensitivity and specificity of 80 and 64 %, respectively.

**Conclusions:**

This study, designed as a proof of principle, indicated that the LDT is an accurate, robust and automated assay, which adequately and reliably identifies patients presenting with small fiber neuropathy, and therefore has potential for use in large scale clinical studies.

## Background

PGP9.5 immunostaining of intra-epidermal nerve fibers in 50 µm sections is widely accepted as the gold standard for the diagnosis of small fiber neuropathy. The assay is used to visualize the number and morphology of the somatic, small caliber, intra-epidermal nerve fibers, and supported by the European Federation of Neurological Societies (EFNS) [1]. The use of skin biopsies as a diagnostic tool in peripheral neuropathies has increased in the last decades, and is regarded as a reliable and standardized tool [1–7]. The Polyneuropathy Task Force [3] concluded that the gold standard was diagnostically efficient at distinguishing polyneuropathy patients [including small fiber neuropathy (SFN)] from normal subjects as controls. Skin biopsy immunostaining can help to detect aberrations in somatic nerves in neuropathies that were formerly classified as autonomic, such as Ross syndrome [8, 9]. It was [4] concluded that the intra-epidermal nerve fiber density (IENF) assessment has proven its value as a measure for treatment success and for follow-up in clinical trials [10–13]. The EFNS Task Force developed guidelines for the diagnostic use of skin biopsies in peripheral neuropathies, published in 2005 [1]. The recommendations include the use of 3-mm punch skin biopsy from the distal leg, fixed in Zamboni solution and quantified for linear nerve fiber density in at least three consecutive 50 µm sections, after PGP9.5 immunohistochemical or immunofluorescence staining. In the counting rules it was emphasized to only include IENF crossing the dermal-epidermal junction, while excluding secondary/tertiary branching from the quantification [1]. From the perspective of use of skin biopsies in clinical trials, the EFNS-advised method is time-consuming and challenging for large scale batch testing in a standardized manner. One way of approaching this challenge is to fully automate the staining procedure.

For each laboratory developed test (LDT), which aims to identify disease and is intended for accreditation and use in clinical trial settings, assay performance characteristics must be established as required by CAP/CLIA [14] and local accreditation boards [15]. Good laboratory practice requires that accuracy, precision, analytical sensitivity, analytical specificity, reportable range and reference intervals are established [16, 17]. Biopsies from subjects clearly showing intra-epidermal nerve fiber reduction, as confirmed by conventional diagnostic tools, were selected from two studies conducted earlier by Ragé and colleagues [18, 19]. In the first study, the experimental model of reversible capsaicin-induced small SFN [10, 11, 20] was examined in healthy subjects using the laser evoked potentials (LEPs) in comparison to the linear nerve fiber density in skin. The second study aimed at investigating the diagnostic performance of LEPs in the assessment of small nerve fiber loss in asymptomatic diabetic neuropathy. From this study, diabetic subjects presenting with distal polyneuropathy were included. In addition, skin biopsies of healthy subjects were studied to indicate achievable reference ranges, and reveal possible discrepancies and quantitative caveats between the LDT and the gold standard method.

In this study we developed an automated PGP9.5 LDT, to complement the gold standard, and validated its potential for use in large scale clinical studies.

## Methods

### Human samples

Fourteen skin punch biopsies are obtained from healthy volunteers (n = 14, age group 33–52 years). Five biopsy specimens, from diabetic subjects presenting poly-neuropathy (n = 5), obtained from the study described by Ragé and coworkers [19] were included in the study. In addition, two biopsy specimens obtained from healthy volunteers who received topical capsaicin application on three consecutive 24-h cycles were examined as well [18]. Studies were approved by the Local Ethics Committee; informed consent was obtained for all participants.

### Skin punch biopsy processing

Skin punch biopsies (diameter 2–4 mm) were performed under local anesthesia at the lateral aspect of the distal leg in a clinical unit. Skin biopsies were fixed in cold Zamboni fixative solution (60 min at room temperature), cryopreserved in sucrose 30 % and frozen in OCT compound (Sakura TissueTek Europe, The Netherlands) as recommended by the EFNS guidelines [1, 21]. After freezing, tissue-blocks were stored at −80 °C prior to sectioning.

### Accuracy confirmation by western blot analysis and double immunofluorescence histochemistry

Western Blot analysis was performed by SDS-page on cell lysates from A549 (lung carcinoma) and U87 (glioblastoma) cell lines exhibiting high PGP9.5 expression, to verify the accuracy of the rabbit polyclonal anti-human PGP9.5 antibody (RA95101, UltraClone Ltd., UK). Subsequently, western transfer of proteins was performed onto a transfer membrane (Immobilon FL, Li-Cor, Germany). After incubation of the rabbit polyclonal anti-human PGP9.5 antibody (1/300), visualization was performed using IRDye^®^ 800CW Conjugated Goat (polyclonal) anti-rabbit IgG antibody on the Odyssey^®^ Infrared Imaging System (Li-Cor).

For double immunofluorescence histochemistry, 16 µm-thick sections were incubated overnight at room temperature with a mix of primary anti-PGP9.5 (1/2000) and anti-βIII-Tubulin (clone 5G8, Promega Corp., USA; 1/250) antibodies to be subsequently visualized by Cy3-conjugated goat anti-rabbit and Alexa-488-conjugated goat anti-mouse antibodies (Jackson Immunoresearch Laboratories, Inc., USA; 1/500 and 1/100 respectively). Counterstain was performed using Hoechst 33342 (Invitrogen Molecular Probes, USA; 1/2000).

### Gold standard PGP9.5 immunofluorescence according to EFNS (GS-EFNS)

Three consecutive 50 µm cryosections were produced and collected in PBS-azide. To enhance penetration of immunoreagents into 50 µm-thick sections, tissue was permeabilized using TritonX100 (Sigma-Aldrich, Belgium; 0.1 %). Manual staining was initiated on free-floating tissue sections. Incubation was performed using the rabbit polyclonal anti-human PGP9.5 primary antibody (UltraClone Ltd.; RA95101; 1/2000) overnight at room temperature as prescribed [1, 22]. Subsequently, the visualization was established using a Cy3-labeled secondary goat anti-rabbit antibody (Jackson Immunoresearch Laboratories; 1/500; 2 h) and counterstained with Hoechst 33342 as described above.

### Automatization of PGP9.5 Immunofluorescence staining on Ventana discovery XT^®^

Six consecutive 16 µm cryosections were produced and collected on dry ice. The staining protocol was custom-programmed using RESEARCH IHC QD Map XT software of the Discovery XT^®^ (Ventana, USA). After loading the slides into the instrument, incubation was performed using the rabbit polyclonal anti-human PGP9.5 primary antibody for 2 h. Subsequently, the visualization was established using a Cy3-labeled secondary goat anti-rabbit antibody and counterstained with Hoechst 33342 as described above.

### Quality control

For all specimens, internal controls, i.e. autonomic nerve fibers innervating sweat glands and m. arrector pili, were evaluated for positive staining and subsequent acceptance of each individual section. As a negative control, rabbit immunoglobulins (confirm negative control rabbit, Ventana) were used to replace the primary antibody.

### Imaging and quantification

The quantification of the linear density of nerve fibers was performed using a conventional fluorescence microscope (20×–40×; Zeiss, Germany) by two readers blinded for treatment. Virtual images were generated using the Axiovision Mozaik Imaging Software (Axiovision Rel 4.8^®^) on an Axioplan 2 imaging microscope equipped with a motorized stage and z-stack features (Zeiss, Germany) and used to perform length measurements. Individual counts were divided by the length of the epidermis and expressed as mean numbers/mm (± SD).

### Gold standard counting rule

The EFNS guidelines [1, 3, 23] prescribe to count each nerve fiber crossing the dermal-epidermal junction as a single unit. Nerve fibers that are approaching the junction without crossing it or nerve fiber fragments lying free in the epidermis are not enumerated, nor are branches.

### LDT modifications to the gold standard counting rule

**Fig. 1:**
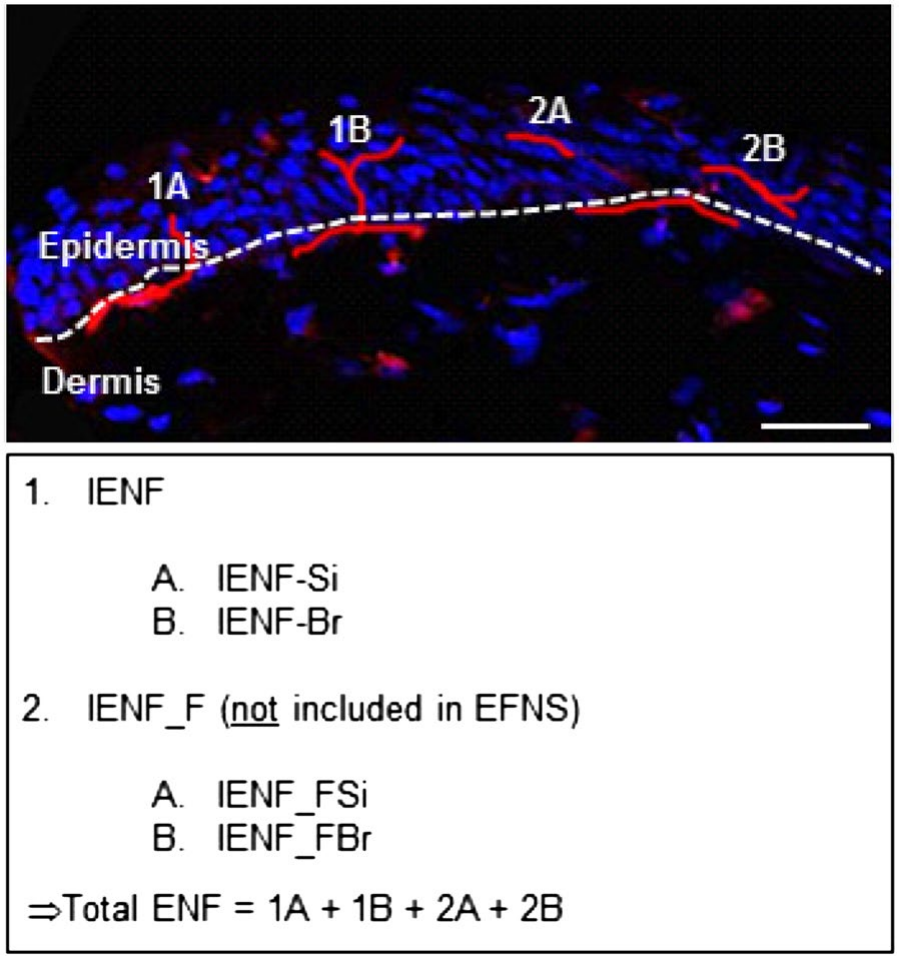
Counting rules according to the GS-EFNS (*1*) and LDT (*2*). Modifications to the gold standard counting rule for intra epidermal nerve fibers (IENF) are depicted to include IENF fragments (_F) detected as single fibers (_Si) or branching (_Br). *Red* PGP9.5, *blue* Hoechst, *dashed line* dermal-epidermal junction. *1* IENF; *1A* IENF_Si; *1B* IENF_Br; *2* IENF_F; *2A* IENF_FSi; *2B* IENF_FBr. (*Scale bar* 20 µm)

To critically evaluate the LDT, and the effect of reducing the section thickness, the following additions to the gold standard counting rule were implemented on both the LDT and gold standard stained slides. The epidermal nerve fibers, traditionally named IENF, include all nerve fibers crossing the dermal—epidermal junction (Fig. 1) as per the gold standard counting rule. In order to accurately define existing discrepancies in the LDTs ability of visualizing the 3D-IENF network, incidence of secondary branching of IENF was taken into account. Therefore, intra-epidermal nerve fiber crossing dermal-epidermal junction as single fiber (IENF_Si) (Fig. 1: *1A*) and intra-epidermal nerve fiber crossing dermal-epidermal junction as fiber showing branching (IENF_Br) (Fig. 1: *1B*) as being epidermal nerve fibers crossing the dermal-epidermal junction as single or branching fibers are reported. Since the LDT uses thinner tissue sections, and a higher incidence of IENF fragments is to be expected, intra-epidermal nerve fiber fragments that do not cross the dermal-epidermal junction free intra-epidermal nerve fiber fragments (IENF_F, Fig. 1: *2A*, *2B*) were included. These fragments were not included in the EFNS guidelines but have been proven to be valuable [24]. Fragments are considered as such based on morphological characteristics and an approximate minimal length of 5 µm. For IENF_F, the incidence of secondary branching of IENF was taken into account as well, resulting in IENF_FSi (Fig. 1: *2A*) and IENF_FBr (Fig. 1: *2B*). The actual number of branches on each nerve fiber was recorded separately. The total number of epidermal nerve fibers (total ENF, Fig. 1) represented the sum of IENF and IENF_F.

### LDT method validation

Besides the confirmation that the anti-human PGP9.5 antibody accurately detects its target, we established reference intervals and defined LDTs discrepancies in reference to the GS-EFNS (LDT modifications to the GS-EFNS counting rule) on biopsies obtained from healthy subjects. Diagnostic yield and diagnostic performance (analytical sensitivity and specificity) were explored by plotting the plausibility of false positives (specificity) and true positives (sensitivity). The closer the ROC curve approaches the true positive axis, the better the performance of the LDT (see “Statistics” section). The examination of the inter-slide stability of the GS-EFNS and LDT was included since this could highlight the need for a minimum number of serial slides to be examined. For each subject and each staining method three serial slide measurements were performed by one observer (M1, M2, and M3). In order to estimate the reliability of results obtained by independent observers, individual counts for the different parameters were compared after automated staining of 12 randomly selected samples.

### Statistics

Method comparison of the LDT and GS-EFNS was performed using Bland–Altman analysis [25]. To prove a good agreement between the two techniques the values should be lumped near the 0-difference line. Statistical analysis was performed using a rank sum test for paired samples (Wilcoxon) for which p values below 0.05 were considered significant. Statistical analysis for assessing the diagnostic yield of the LDT compared to conventional diagnostic tools was performed as described before [19]. To explore if data obtained using the EFNS advised method can serve as the gold standard to define the diagnostic performance of the LDT on the selected biopsies, a one-way analysis of variance was performed (ANOVA). This allowed confirming that mean values were significantly different between control and SFN groups. The diagnostic performance of the LDT was estimated by the area under the receiver operating characteristic curve (ROC) with 95 % confidence interval for sensitivity and specificity using De Long’s test. Inter-observer agreement was evaluated by determining intra-class correlation (ICC) for all parameters using the same raters for all measurements and consistency as type. All analyses were performed using MedCalc^®^ v12.3.0.0 statistical software. Finally, a power calculation was carried out to determine the statistical power of this study and the optimal sample size for a future study using software package R, version 3.1.2 [26]. We performed this analysis using data for the total linear density of epidermal nerve fibers.

## Results

### Accuracy of the anti-human PGP9.5 antibody

**Fig. 2:**
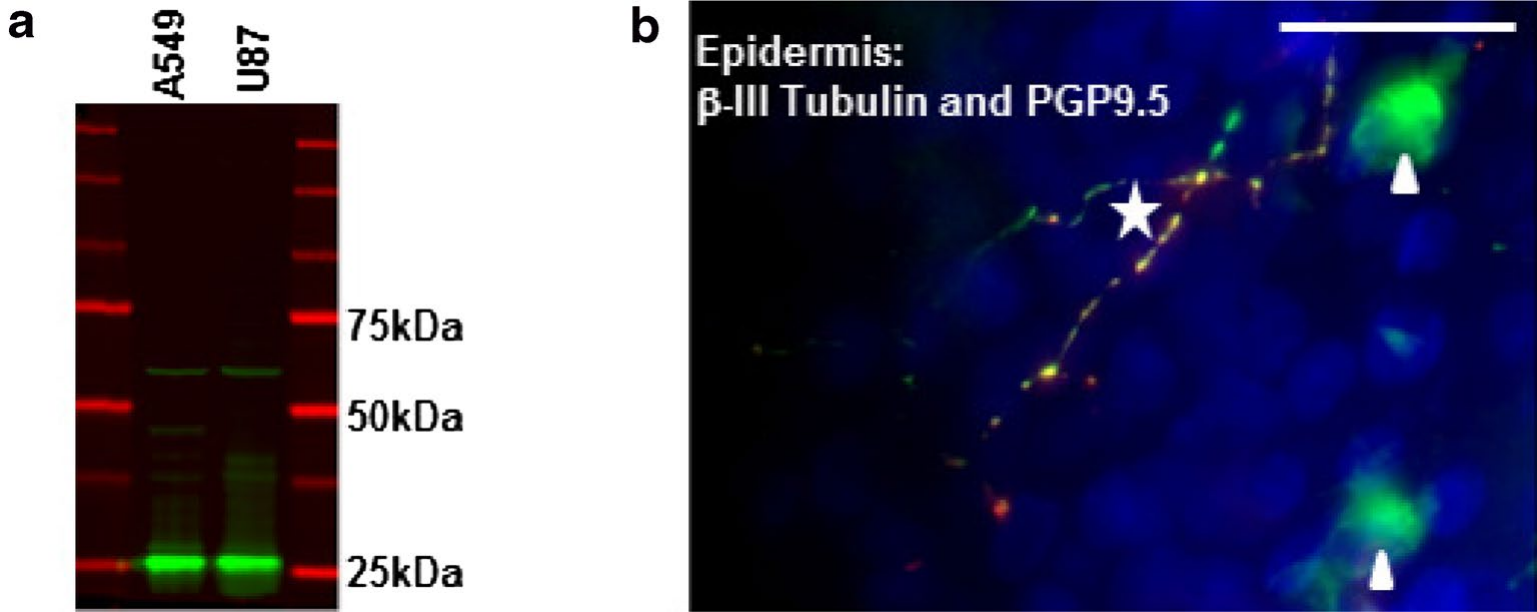
Evaluation of antibody accuracy. **a** Western Blot analysis of A549 and U87 cell lines using the rabbit polyclonal anti-human PGP9.5 antibody (*green*). **b** Immunofluorescent staining of PGP9.5 (*red*) and ß-III-Tubulin (*green*) in the epidermis of a human skin biopsy, *blue* Hoechst. *Asterisk* Nerve fiber; *Arrow head* Langerhans-cells. (*Scale bar* 20 µm)

The accuracy of the rabbit polyclonal anti-human PGP9.5 antibody was confirmed by western blot analysis on cell lysates from U87 and A549 cell lines. For both cell lines the antibody showed a band at a molecular weight of approximately 25 kDa (Fig. 2a), confirming the recognition of PGP9.5 (27 kDa). In addition, βIII-tubulin, a nerve and langerhans cell-specific marker, co-localized in all PGP9.5 immunoreactive structures of the epidermis (Fig. 2b), confirming the accuracy of the antibody.

### Assessment of IENF and nerve fiber branching density in skin biopsies of healthy volunteers**

**Fig. 3:**
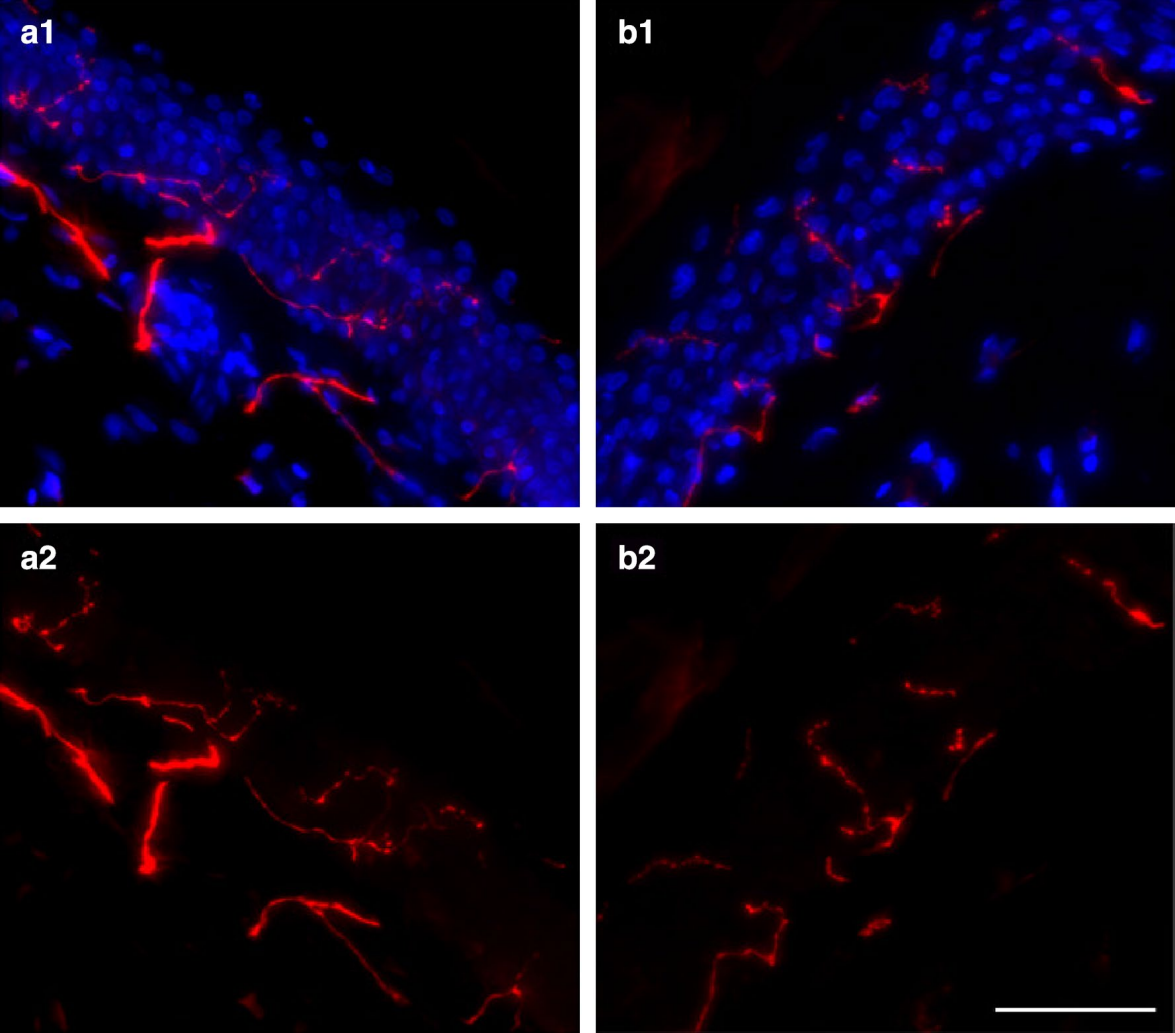
PGP9.5 Immunofluorescence in skin biopsy of a healthy volunteer. PGP9.5 immunofluorescence using the gold standard (GS-EFNS: **a1**) method and laboratory developed test (LDT: **b1**) was performed as described. **a2** and **b2** show the single channel image of PGP9.5 immunofluorescence (red). *Red* PGP9.5, *Blue* Hoechst. (*Scale bar* 50 µm)

A significant difference existed in the ability of the LDT assessing IENF compared to the gold standard (p < 0.001, mean difference 5.8 IENF/mm), especially with regard to IENF_Br (p < 0.001, mean difference 5.7 IENF_Br/mm) (Table 1). For IENF_Si equal results were obtained for the GS-EFNS and LDT (p = 0.95, mean difference 0.1 IENF_Si/mm). Enumeration of nerve fiber fragments lying free in the epidermis showed a significantly higher number of IENF_F for the LDT compared to GS-EFNS (p = 0.01, mean difference −2.8 IENF_F/mm). The IENF_F were mainly present as single nerve fiber fragments (IENF_FSi; p = 0.002, mean difference −2.5 IENF_FSi/mm). Overall, for the total population of epidermal nerve fibers (IENF and IENF_F) a good agreement was reached between the GS-EFNS and LDT (p = 0.24, mean difference 3.0 Total ENF/mm) (Fig. 3; Table 1). Based on the mean difference and SD of both measurements, we determined that the difference between the groups equals 0.68 SD units. A power calculation showed that, to detect a difference in means of 0.68 SD units, with a power of 80 %, at alpha level of 0.05, sample size should be at least 35 in both groups.

**Table 1:** Comparing GS-EFNS and LDT for assessing nerve fiber density in skin biopsies of healthy volunteers

	GS-EFNS	LDT	Mean diff	P
*IENF (#/mm)*	11.47 ± 2.67	5.65 ± 1.93	5.8	<0.001
IENF_Si (#/mm)	4.45 ± 1.06	4.31 ± 1.59	0.1	0.95
IENF_Br (#/mm)	7.02 ± 2.23	1.34 ± 0.51	5.7	<0.001
Sec branching (#/IENF_Br)	3.17 ± 0.26	2.39 ± 0.40		<0.001
Sec branching (#/mm)	22.04 ± 7.92	3.56 ± 1.44	18.5	<0.001
*IENF_F (#/mm)*	3.98 ± 2.25	6.77 ± 2.89	−2.8	0.01
IENF_FSi (#/mm)	2.36 ± 1.10	4.86 ± 2.18	−2.5	0.002
IENF_FBr (#/mm)	1.62 ± 1.28	1.91 ± 0.86	−0.3	0.36
Sec branching (#/IENF_FBr)	2.52 ± 0.57	2.13 ± 0.23		0.04
Sec branching (#/mm)	4.42 ± 3.91	4.26 ± 1.88		0.01
*Total ENF (#/mm)*	15.45 ± 4.43	12.45 ± 4.33	3.0	0.24

*ENF* epidermal nerve fiber, *GS-EFNS* gold standard method according to EFNS; *IENF* intra-epidermal nerve fiber, *IENF_Si* intra-epidermal nerve fiber crossing dermal-epidermal junction as single fiber, *IENF_Br* intra-epidermal nerve fiber crossing dermal-epidermal junction as fiber showing branching, *IENF_F* nerve fiber fragment lying free in epidermis, *IENF_FSi* nerve fiber fragment lying free in epidermis as single fragment, *IENF_FBr* nerve fiber fragment lying free in epidermis showing branching, *LDT* laboratory developed test, *Sec* secondary

A significantly lower number of IENF branches was observed using the LDT (p < 0.001, mean difference 18.5 Branches/mm). Minor differences were seen for IENF fragment branches (p = 0.04, mean difference 0.2 Branches/mm) (Fig. 3; Table 1).

### Diagnostic performance of the LDT for SFN

**Fig. 4:**
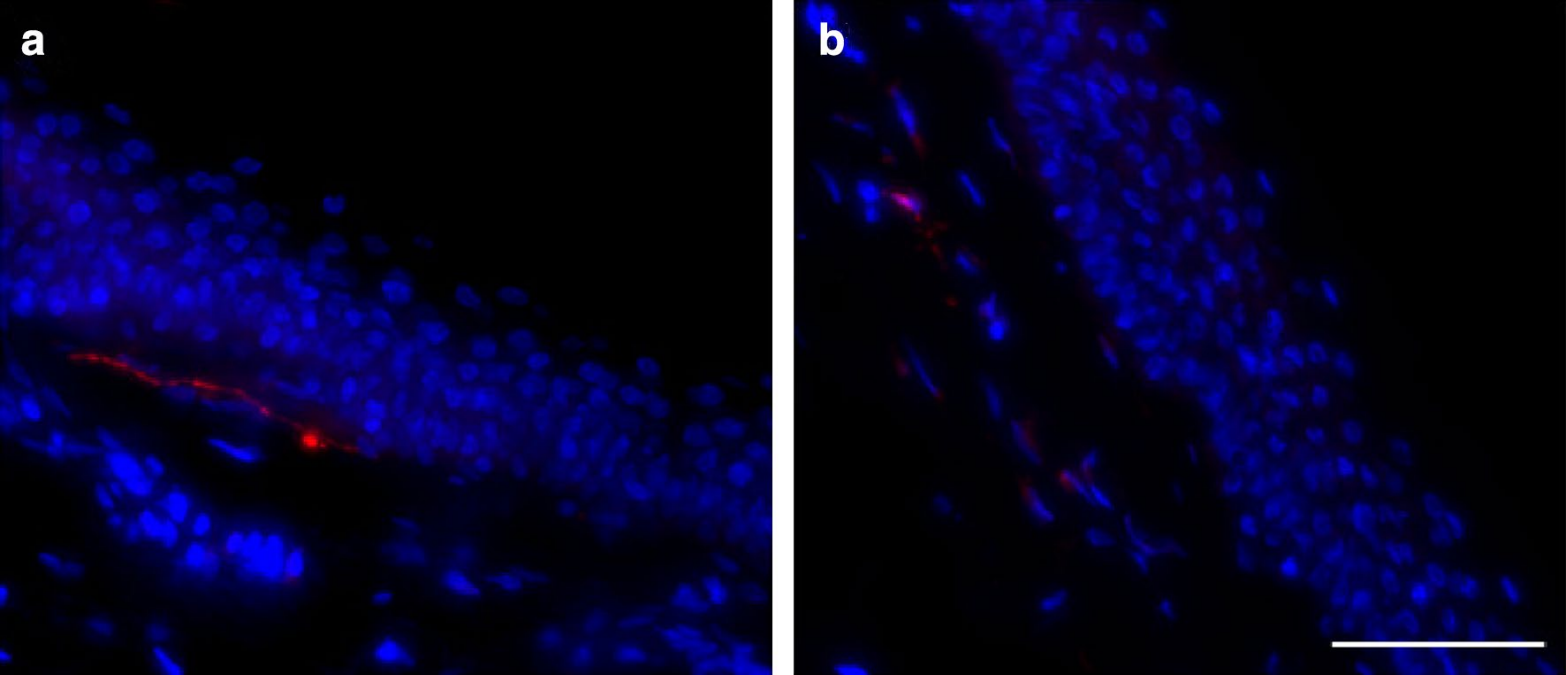
PGP9.5 Immunofluorescence in skin biopsy of a SFN patient. PGP9.5 immunofluorescence using the gold standard (GS-EFNS: **a**) and laboratory developed test (LDT: **b**) was performed as described. *Red* PGP9.5, *Blue* Hoechst. (*Scale bar* 50 µm)

The LDT was able to distinguish between healthy and SFN groups (p < 0.001) when assessing IENF linear density (Table 2). Importantly, the LDT reached good concordance with the gold standard as determined by Bland–Altman after assessing IENF (p > 0.42, mean difference 0.23 IENF/mm); IENF_F (p > 0.25, mean difference −0.29 IENF/mm) and thus total ENF (p > 0.42, mean difference −0.06 ENF/mm) in skin biopsies of SFN subjects. This clearly indicates that the LDT and GS-EFNS are equivalent in detecting SFN (Fig. 4). A lower number of nerve fiber fragments lying free in the epidermis (IENF_F) was observed when the gold standard was applied (Table 2).

**Table 2:** Comparing GS-EFNS and LDT for assessing nerve fiber density in skin biopsies of SFN patients

	GS-EFNS	LDT	Mean diff.	P
*IENF*	1.12 ± 1.65	0.89 ± 1.35	0.23	0.42
IENF_Si	0.55 ± 0.56	0.71 ± 0.95	−0.16	0.42
IENF_Br	0.57 ± 1.16	0.18 ± 0.43	0.39	0.09
*IENF_F*	0.38 ± 0.84	0.66 ± 1.16	−0.29	0.25
IENF_FSi	0.23 ± 0.49	0.46 ± 0.76	−0.23	0.30
IENF_FBr	0.15 ± 0.35	0.21 ± 0.41	−0.06	0.17
*Total ENF*	1.50 ± 2.46	1.56 ± 2.48	−0.06	0.42

*Diff* difference, *ENF* epidermal nerve fiber, *GS-EFNS* gold standard method according to EFNS, *IENF* intra-epidermal nerve fiber, *IENF_Si* intra-epidermal nerve fiber crossing dermal-epidermal junction as single fiber, *IENF_Br* intra-epidermal nerve fiber crossing dermal-epidermal junction as fiber showing branching, *IENF_F* nerve fiber fragment lying free in epidermis, *IENF_FSi* nerve fiber fragment lying free in epidermis as single fragment, *IENF_FBr* nerve fiber fragment lying free in epidermis showing branching, *LDT* laboratory developed test

When secondary branching of nerve fibers was taken into account, a good agreement was found for all parameters evaluated, showing mean differences less than 0.5/mm. Nevertheless, lower scoring for IENF without secondary branching (mean difference of −0.16 IEFN_Si/mm) (p = 0.42) at one hand and a higher assessment of IENF showing secondary branching (mean difference of 0.39 IENF_Br/mm) (p = 0.09) was seen using the gold standard, as was the case in the biopsies from the healthy volunteers (Table 2).

### Diagnostic yield of the LDT for SFN

**Fig. 5:**
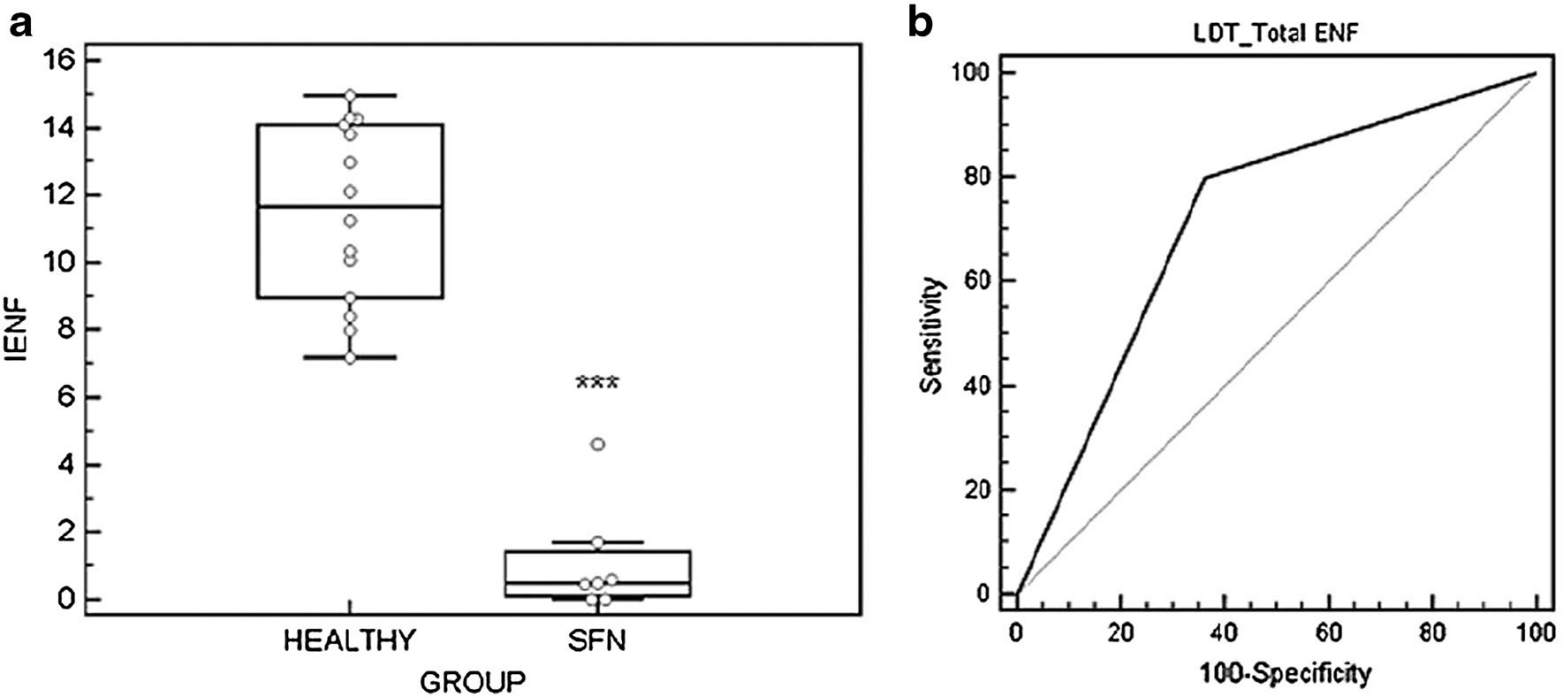
Diagnostic performance of the LDT for SFN. **a** Multiple comparisons graphic, retrieved after one-way analysis of variance (ANOVA) of IENF data obtained using gold standard staining (GS-EFNS) of skin biopsies from healthy volunteers and SFN patients (*** p < 0.001). **b** Diagnostic performance of LDT for variable Total ENF/mm: area under the ROC curve of 0.72 and p = 0.031 with a sensitivity of 80 % and specificity of 64 %. The lower average Total ENF linear density value in the tested control group, 11.02 Total ENF/mm, was used as cut-off

ANOVA analysis confirmed that GS-EFNS can serve as gold standard for the diagnosis of SFN when performed in our lab. Data were grouped using the knowledge of the disease states of the different subjects. A significant difference was observed for IENF between healthy and SFN groups (p < 0.001, F-ratio 87.5) (Fig. 5a). Therefore, GS-EFNS results could be used to determine the expected diagnosis to be obtained using the LDT (ROC, Fig. 5b).

As IENF density, determined using the LDT, is significantly lower compared to the GS-EFNS, the total population of epidermal nerve fibers counted (Total ENF/mm) was used as cut-off. The average linear density in the tested control group was 15.45 ± 4.43 Total ENF/mm (Table 1), therefore the lower cut-off used was 11.02 Total ENF/mm. Using this cut-off to classify data obtained with the LDT, a sensitivity of 80 % and specificity of 64 % was reached with an area under the ROC curve of 0.72 and p = 0.031.

### Inter-slide stability and robustness

For all parameters and both staining methods, mean differences between repeated measures (M1, 2, 3) observed were very small, not exceeding 1 nerve fiber/mm as determined by Bland–Altman analysis (Table 3). Both techniques showed a success rate exceeding 95 % with no failure and thus no exclusion of samples when the LDT is applied.

**Table 3:** Assessment of the inter-slide stability for scoring PGP9.5 using Bland–Altman analysis for the comparison of GS-EFNS and LDT

	Mean diff (#/mm)
*GS-EFNS*	
M1–M2	
IENF_Si	−0.3
IENF_Br	−0.3
IENF_FSi	0.2
IENF_FBr	0.4
IENF_Si	−0.3
M1–M3	
IENF_Si	0.2
IENF_Br	0.5
IENF_FSi	0.5
IENF_FBr	0.2
*LDT*	
M1–M2	
IENF_Si	−0.9
IENF_Br	0
IENF_FSi	−0.3
IENF_FBr	−0.2
M1–M3	
IENF_Si	−0.8
IENF_Br	0.5
IENF_FSi	−0.6
IENF_FBr	0.2

*Diff* difference, *ENF* epidermal nerve fiber, *GS-EFNS* gold standard method according to EFNS, *IENF* intra-epidermal nerve fiber, *IENF_Si* intra-epidermal nerve fiber crossing dermal-epidermal junction as single fiber, *IENF_Br* intra-epidermal nerve fiber crossing dermal-epidermal junction as fiber showing branching, *IENF_F* nerve fiber fragment lying free in epidermis, *IENF_FSi* nerve fiber fragment lying free in epidermis as single fragment, *IENF_FBr* nerve fiber fragment lying free in epidermis showing branching, *LDT* laboratory developed test

### Inter-observer agreement

Overall, the inter-reader agreement can be considered excellent with ICC values ranging from 0.93 to 0.99 (Table 4) for all linear density values enumerated on LDT stained samples.

**Table 4:** Inter-observer agreement when determining nerve fiber density in skin biopsies of SFN patients using LDT

	ICC
*IENF*	0.97
IENF_Si	0.98
IENF_Br	0.93
*IENF_F*	0.99
IENF_FSi	0.99
IENF_FBr	0.97
*Total ENF*	0.99

*ENF* epidermal nerve fiber *GS-EFNS*: gold standard method according to EFNS, *ICC* itra-class correlation, *IENF* intra-epidermal nerve fiber, *IENF_Si* intra-epidermal nerve fiber crossing dermal-epidermal junction as single fiber, *IENF_Br* intra-epidermal nerve fiber crossing dermal-epidermal junction as fiber showing branching, *IENF_F* ierve fiber fragment lying free in epidermis, *IENF_FSi* ierve fiber fragment lying free in epidermis as single fragment, *IENF_FBr* nerve fiber fragment lying free in epidermis showing branching, *LDT* laboratory developed test, *SFN* small fiber neuropathy

## Discussion

As proven by multiple investigators, skin biopsies are excellent tools to investigate the nerve fiber endings in the epidermis [1–7]. Since the early nineties, the neuronal biomarker PGP9.5 has been regarded as the most accurate for the visualization of epidermal nerves [22, 27]. As the use of this technique grew for the diagnosis of SFN, the need for guidelines and standardization grew accordingly. In 2005, the members of the EFNS indicated the advised procedure for the assessment of epidermal nerve fiber density [1]. The gold standard was described in detail [23], and implemented successfully in specialized laboratories over the world. Other investigators used the IENF density as a reference in experimental disease progression studies [10, 11], and early detection of SFN in diabetics [12, 13], and compared its accuracy to that of conventional diagnostic tools. Nevertheless, this method is a labor intense, manual staining procedure, requiring high methodological skills and training, and is therefore prone to human error when applied in conventional diagnostic laboratories. The use of uniform outcome measures in peripheral neuropathies has been commended recently, to enable comparison between studies [28].

In this study, we explored whether a gold standard-related LDT could be developed, with decreased labor and increase of standardization as primary goals, by automating the staining method. Automated slide staining use implied a significant decrease in section thickness of the skin biopsies. The choice of using 16 µm-thick sections was driven by the work of Torres and colleagues [29] and Hedreen [30], both describing that thicker sections mounted on glass slides can present suboptimal penetration of immuno-reagents into the tissue, leading to a lost-cap phenomenon. The use of thinner sections for subsequent IENF assessment has been proven valuable by different groups, not only for SFN detection, but also the study of multiple nerve markers including ion channels and receptors in the same biopsies and the progression and/or follow up of disease [6, 22, 31–36].

The design of the LDT and its subsequent validation was based on requirements of CAP, CLIA and a local accreditation board (BELAC) to be able to introduce the LDT in large scale batch testing required for clinical trial testing. We explored whether the LDT could meet the needs for both an accredited laboratory and the pharmaceutical industry, while maintaining a good concordance with the gold standard. In this study, fluorescence detection was preferred to allow future advanced imaging as described recently [37, 38]. Published data showing good concordance between fluorescent and bright-field assessment of IENF in the diagnosis of SFN justifies this choice [39]. Implementation of fluorescence in IENF assessment additionally helps with the ease of studying multiple targets in a single tissue section.

Accuracy of the PGP9.5 antibody was confirmed in our hands, being concordant with results described by groups of Brichory, Krimm, Tokumaru and Barrachina [40–43].

Once good accuracy was confirmed, the automated user-defined assay performed on the Discovery XT^®^ was challenged against the gold standard using skin biopsies of healthy volunteers. We first demonstrated that performance of the assay in our accredited laboratory according the EFNS advised gold standard method meets all criteria. A good concordance was reached with published normative values (9.8–12.4 IENF/mm for female subjects in the age group of 33–52) as described by Lauria and co-workers [23] when assessing IENF in skin biopsies of predominantly female subjects included in this study. The gold standard could distinguish between healthy and SFN subjects with high significance and served as a reference.

When exploring the IENF density as assessed by the LDT, results clearly indicated that the LDT’s ability to visualize IENF in skin of healthy subjects is considerably lower compared to the gold standard, whereas a good concordance was reached when both IENF and IENF fragments were considered (total ENF). A higher number of IENF and visualization of their branches using the gold standard is in line with the increased occurrence of nerve fiber fragments observed in the thinner sections of the LDT. Considering the threefold decrease in section thickness, one would expect to have a threefold decrease in linear densities of the evaluated parameters. As we found an approximate twofold decrease for healthy subjects and 1.25-fold decrease in SFN subjects compared to the GS, one must consider that nerve fibers might be included more than once in the counting strategy when the LDT is applied. Nevertheless, the diagnostic performance of the LDT was equal to that of the gold standard in discriminating healthy from SFN subjects with high significance when IENF was assessed.

Additionally, an excellent agreement between both staining methods was found for all parameters quantified in biopsies from subjects with (experimental) SFN. Both the number of IENF, IENF fragments and their secondary branching could be equally visualized. In addition, previously published work documented concordance of the LDT’s outcome with TRPV1 staining performed in a different laboratory. Results describing a functional recovery, prior to morphological recovery in a time course study after capsaicin was applied, were consistent with findings of other groups [18].

When the diagnostic yield of the LDT was measured, we concluded that IENF and IENF fragments are both mandatory to be included in enumeration when the LDT is applied. IENF assessment alone lacked concordance with the gold standard method. When the total number of nerve fibers (IENF and IENF fragments) was considered, the LDT reached relatively good concordance with the gold standard, with strong sensitivity and specificity. Finally, the LDT showed an excellent robustness and an excellent inter-observer concordance.

## Conclusions

To conclude, in this proof of principle study we evaluated a standardized, on-slide, automated staining procedure for the assessment of the linear density of epidermal nerve fibers. This method demonstrated an equal performance to the gold standard in distinguishing SFN from healthy subjects. By automation and on-slide application of staining thinner sections, labor, variation of results induced by human manipulation and differential penetration of immunoreagents are drastically reduced. The decreased sensitivity, however, for detecting the complex three-dimensional branching network of epidermal nerve fibers is important to consider, as depletion of more distal axons is likely to be an early feature of dying back neuropathies. In this perspective, the appearance of collateral sprouting, typical for regenerating nerve fibers [44], and the LDT’s capability of discriminating that from normal epidermal nerve fibers, needs further investigation. The same applies for the risk of missing the SFN status in older patients. Effectiveness in detection of important cutaneous nerve abnormalities, such as axonal swellings, crawlers and sprouts, by the LDT need to be examined as well [45]. Formation of a larger cohort, minimally 35 per group as determined by power calculation and inclusion of a solid age and gender distribution is required to determine reference values and to determine whether the dynamic range of the LDT is acceptable. Power calculation showed that the current study design only offers 29 % power to detect a difference in means of the calculated SD units. Since epidermal nerve fiber fragments need to be included in the now more laborious counting strategy, compatibility of this technique with the semi-automated analysis recently presented [38] should be investigated as well. This technique could lead to high reproducibility of counting within and between neuropathological institutes. Introducing proficiency testing and dermal nerve quantification [46] could benefit in standardization.

Full automation of the staining procedure could be valuable and lead to accessible, stable and reliable testing in clinical trials and diagnostics for clear SFN detection. A number of limitations however exist for this LDT which require expert-evaluation in a larger cohort.

## Abbreviations

ANOVA: analysis of variation
BELAC: Belgium’s accreditation organization
CAP/CLIA: College of American Pathologists/Clinical Laboratory Improvement Act
ENF: epidermal nerve fiber
EFNS: European Federation of Neurological Societies
GS: gold standard
GS-EFNS: gold standard method according to EFNS
ICC: intra-class correlation
IENF: intra-epidermal nerve fiber density
IENF_F: free intra-epidermal nerve fiber fragments
IENF_Si: intra-epidermal nerve fiber crossing dermal-epidermal junction as single fiber
IENF_Br: intra-epidermal nerve fiber crossing dermal-epidermal junction as fiber showing branching
IENF_FSi: nerve fiber fragment lying free in epidermis as single fragment
IENF_FBr: nerve fiber fragment lying free in epidermis showing branching
LDT: laboratory developed test
LEPs: laser evoked potentials
OCT: optimal cutting temperature
PBS: phosphate buffered saline
ROC: receiver operator characteristics
SD: standard deviation
SDS: sodium dodecyl sulphate
SNF: small fiber neuropathy

## References

[1] Lauria G, Cornblath DR, Johansson O, McArthur JC, Mellgren SI, Nolano M, Rosenberg N, Sommer C (2005). EFNS guidelines on the use of skin biopsy in the diagnosis of peripheral neuropathy. Eur J Neurol.

[2] Hlubocky A, Wellik K, Ross MA, Smith BE, Hoffman-Snyder C, Demaerschalk BM, Wingerchuk DM (2010). Skin biopsy for diagnosis of small fiber neuropathy: a critically appraised topic. Neurologist.

[3] England JD, Gronseth GS, Franklin G, Carter GT, Kinsella LJ, Cohen JA, Asbury AK, Szigeti K, Lupski JR, Latov N, Lewis RA, Low PA, Fisher MA, Herrmann D, Howard JF, Lauria G, Miller RG, Polydefkis M, Sumner AJ. Practice parameter: the evaluation of distal symmetric polyneuropathy: the role of autonomic testing, nerve biopsy, and skin biopsy (an evidence-based review) report of the American Academy of Neurology, the American Association of Neuromuscular and Elec. PM R 200;1:14–22.

[4] Sommer C (2008). Skin biopsy as a diagnostic tool. Curr Opin Neurol.

[5] Hsieh S-T (2006). EFNS guidelines on the use of skin biopsy in the diagnosis of peripheral neuropathy. Eur J Neurol.

[6] Koskinen M, Hietaharju A, Kyläniemi M, Peltola J, Rantala I, Udd B, Haapasalo H (2005). A quantitative method for the assessment of intraepidermal nerve fibers in small-fiber neuropathy. J Neurol.

[7] Nolano M, Provitera V, Crisci C, Stancanelli A, Wendelschafer-Crabb G, Kennedy WR, Santoro L (2003). Quantification of myelinated endings and mechanoreceptors in human digital skin. Ann Neurol.

[8] Perretti A, Nolano M, De Joanna G, Tugnoli V, Iannetti G, Provitera V, Cruccu G, Santoro L (2003). Is Ross syndrome a dysautonomic disorder only? an electrophysiologic and histologic study. Clin Neurophysiol..

[9] Sommer C, Lauria G (2007). Skin biopsy in the management of peripheral neuropathy. Lancet Neurol.

[10] Nolano M, Simone DA, Wendelschafer-Crabb G, Johnson T, Hazen E, Kennedy WR (1999). Topical capsaicin in humans: parallel loss of epidermal nerve fibers and pain sensation. Pain.

[11] Polydefkis M, Hauer P, Sheth S, Sirdofsky M, Griffin JW, McArthur JC (2004). The time course of epidermal nerve fibre regeneration: studies in normal controls and in people with diabetes, with and without neuropathy. Brain.

[12] Umapathi T, Tan WL, Loke SC, Soon PC, Tavintharan S, Chan YH (2007). Intraepidermal nerve fiber density as a marker of early diabetic neuropathy. Muscle Nerve.

[13] Løseth S, Stålberg E, Jorde R, Mellgren SI (2008). Early diabetic neuropathy: thermal thresholds and intraepidermal nerve fibre density in patients with normal nerve conduction studies. J Neurol.

[14] College of American Pathologists: Anatomic Pathology Checklist. CAP lab accredit checklists. http//www.cap.org/apps/cap.portal. Accessed May 2014, 25 Sept 2012. 1–42.

[15] BELAC: NBN EN ISO 15189. 2012, 03.120.10(September):149.

[16] Fitzgibbons PL, Bradley LA, Fatheree LA, Alsabeh R, Fulton RS, Goldsmith JD, Haas TS, Karabakhtsian RG, Loykasek PA, Marolt MJ, Shen SS, Smith AT, Swanson PE (2014). Principles of analytic validation of immunohistochemical assays: guideline from the College of American Pathologists Pathology and Laboratory Quality Center. Arch Pathol Lab Med..

[17] Howat WJ, Lewis A, Jones P, Kampf C, Pontén F, van der Loos CM, Gray N, Womack C, Warford A (2014). Antibody validation of immunohistochemistry for biomarker discovery: recommendations of a consortium of academic and pharmaceutical based histopathology researchers. Methods.

[18] Ragé M, VanAcker N, Facer P, Shenoy R, Knaapen MWM, Timmers M, Streffer J, Anand P, Meert T, Plaghki L (2010). The time course of CO(2) laser-evoked responses and of skin nerve fibre markers after topical capsaicin in human volunteers. Clin Neurophysiol..

[19] Ragé M, Van Acker N, Knaapen MWM, Timmers M, Streffer J, Hermans MP, Sindic C, Meert T, Plaghki L (2011). Asymptomatic small fiber neuropathy in diabetes mellitus: investigations with intraepidermal nerve fiber density, quantitative sensory testing and laser-evoked potentials. J Neurol.

[20] Carpenter SE, Lynn B (1981). Vascular and sensory responses of human skin to mild injury after topical treatment with capsaicin. Br J Pharmacol.

[21] Lauria G, Bakkers M, Schmitz C, Lombardi R, Penza P, Devigili G, Smith AG, Hsieh ST, Mellgren SI, Umapathi T, Ziegler D, Faber CG, Merkies ISJ (2010). Intraepidermal nerve fiber density at the distal leg: a worldwide normative reference study. J Peripher Nerv Syst.

[22] Wang L, Hilliges M, Jernberg T, Wiegleb-Edström D, Johansson O (1990). Protein gene product 9.5-immunoreactive nerve fibres and cells in human skin. Cell Tissue Res.

[23] Lauria G, Hsieh ST, Johansson O, Kennedy WR, Leger JM, Mellgren SI, Nolano M, Merkies ISJ, Polydefkis M, Smith AG, Sommer C, Valls-Solé J (2010). European Federation of Neurological Societies/Peripheral Nerve Society guideline on the use of skin biopsy in the diagnosis of small fiber neuropathy. Report of a joint task force of the European Federation of Neurological Societies and the Peripheral Ner. Eur J Neurol.

[24] Ebenezer GJ, Hauer P, Gibbons C, McArthur JC, Polydefkis M (2007). Assessment of epidermal nerve fibers: a new diagnostic and predictive tool for peripheral neuropathies. J Neuropathol Exp Neurol.

[25] Bland JM, Altman DG (1986). Statistical methods for assessing agreement between two methods of clinical measurement. Lancet.

[26] R Core Team. R: A language and environment for statistical computing. Vienna: R Foundation for statistical computing; 2014. http://www.R-project.org/

[27] Dalsgaard CJ, Rydh M, Haegerstrand A (1989). Cutaneous innervation in man visualized with protein gene product 9.5 (PGP 9.5) antibodies. Histochemistry.

[28] Merkies ISJ, Faber CG, Lauria G (2015). Advances in diagnostics and outcome measures in peripheral neuropathies. Neurosci Lett.

[29] Torres EM, Meldrum A, Kirik D, Dunnett SB (2006). An investigation of the problem of two-layered immunohistochemical staining in paraformaldehyde fixed sections. J Neurosci Methods.

[30] Hedreen JC (1998). Lost caps in histological counting methods. Anat Rec.

[31] Malmberg AB, Mizisin AP, Calcutt NA, von Stein T, Robbins WR, Bley KR (2004). Reduced heat sensitivity and epidermal nerve fiber immunostaining following single applications of a high-concentration capsaicin patch. Pain.

[32] Atherton DD, Facer P, Roberts KM, Misra VP, Chizh BA, Bountra C, Anand P (2007). Use of the novel contact heat evoked potential stimulator (CHEPS) for the assessment of small fibre neuropathy: correlations with skin flare responses and intra-epidermal nerve fibre counts. BMC Neurol.

[33] Beiswenger KK, Calcutt NA, Mizisin AP (2008). Epidermal nerve fiber quantification in the assessment of diabetic neuropathy. Acta Histochem.

[34] Thomsen NOB, Englund E, Thrainsdottir S, Rosén I, Dahlin LB (2009). Intraepidermal nerve fibre density at wrist level in diabetic and non-diabetic patients. Diabet Med.

[35] Narayanaswamy H, Facer P, Misra VP, Timmers M, Byttebier G, Meert T, Anand P (2012). A longitudinal study of sensory biomarkers of progression in patients with diabetic peripheral neuropathy using skin biopsies. J Clin Neurosci.

[36] Vencappa S, Donaldson LF, Hulse RP (2015). Original article cisplatin induced sensory neuropathy is prevented by vascular endothelial growth factor-A.. Am J Transl Res.

[37] Provitera V, Nolano M, Stancanelli A, Caporaso G, Vitale DF, Santoro L (2015). Intraepidermal nerve fiber analysis using immunofluorescence with and without confocal microscopy. Muscle Nerve.

[38] Seger S, Stritt M, Doppler K, Frank S, Panaite A, Kuntzer T, Steck A, Pagenstecher A, Sommer C, Stalder AK. A semi-automated method to assess intraepidermal nerve fibre density in human skin biopsies. Histopathology .2015 10.1111/his.12794 26249211

[39] Nolano M, Biasiotta A, Lombardi R, Provitera V, Stancanelli A, Caporaso G, Santoro L, Merkies ISJ, Truini A, Porretta-Serapiglia C, Cazzato D, Dacci P, Vitale DF, Lauria G (2015). Epidermal innervation morphometry by immunofluorescence and bright-field microscopy. J Peripher Nerv Syst.

[40] Brichory F, Beer D, Le Naour F, Giordano T, Hanash S (2001). Proteomics-based identification of protein gene product 9.5 as a tumor antigen that induces a humoral immune response in lung cancer. Cancer Res.

[41] Barrachina M, Moreno J, Juvés S, Moreno D, Olivé M, Ferrer I (2007). Target genes of neuron-restrictive silencer factor are abnormally up-regulated in human myotilinopathy. Am J Pathol.

[42] Tokumaru Y, Yamashita K, Kim MS, Park HL, Osada M, Mori M, Sidransky D (2008). The role of PGP9.5 as a tumor suppressor gene in human cancer. Int J Cancer.

[43] Krimm RF, Davis BM, Noel T, Albers KM (2006). Overexpression of neurotrophin 4 in skin enhances myelinated sensory endings but does not influence sensory neuron number. J Comp Neurol.

[44] Lauria G, Devigili G (2007). Skin biopsy as a diagnostic tool in peripheral neuropathy. Nat Clin Pract Neurol.

[45] Wendelschafer-Crabb G, Kennedy WR, Walk D (2006). Morphological features of nerves in skin biopsies. J Neurol Sci.

[46] Schaumburg H, Arezzo J, Lauria G, Faber CG, Merkies ISJ (2011). Morphometry of dermal nerve fibers in human skin. Neurology..

